# Purification and Functional Characterization of the Chloroform/Methanol-Soluble Protein 3 (CM3) From *Triticum aestivum* in *Drosophila melanogaster*

**DOI:** 10.3389/fnut.2020.607937

**Published:** 2020-12-23

**Authors:** Anna-Lena Thiel, Mohab Ragab, Anika E. Wagner, Senad Divanovic, Stefanie Derer, Christian Sina

**Affiliations:** ^1^Institute of Nutritional Medicine, Molecular Gastroenterology, University Hospital Schleswig-Holstein, Campus Lübeck, Lübeck, Germany; ^2^Institute of Nutritional Sciences, Nutrition and Immune System, Justus-Liebig University Giessen, Giessen, Germany; ^3^Department of Pediatrics, University of Cincinnati College of Medicine, Cincinnati, OH, United States; ^4^Division of Immunobiology, Cincinnati Children's Hospital Medical Center, Cincinnati, OH, United States; ^5^Center for Inflammation and Tolerance, Cincinnati Children's Hospital Medical Center, Cincinnati, OH, United States; ^6^Institute of Nutritional Medicine and 1st Department of Medicine, Section of Nutritional Medicine, University Hospital Schleswig-Holstein, Campus Lübeck, Lübeck, Germany

**Keywords:** CM3, ATIs, α-glucosidase, non-celiac wheat sensitivity, *Drosophila melanogaster*

## Abstract

Non-celiac wheat sensitivity (NCWS) has been proposed to be an independent disease entity that is characterized by intestinal (e.g., abdominal pain, flatulence) and extra-intestinal symptoms (e.g., headache, fatigue), which are propagated following the ingestion of wheat products. Increased activity of amylase trypsin inhibitors (ATIs) in modern wheat is suggested to be major trigger of NCWS, while underlying mechanisms still remain elusive. Here, we aimed to generate and functionally characterize the most abundant ATI in modern wheat, chloroform/methanol-soluble protein 3 (CM3), *in vitro* and in *Drosophila melanogaster*. We demonstrate that CM3 displays α-glucosidase but not α-amylase or trypsin inhibitory activity *in vitro*. Moreover, fruit flies fed a sucrose-containing diet together with CM3 displayed significant overgrowth of intestinal bacteria in a sucrose-dependent manner while the consumption of α-amylase and α-glucosidase inhibitors was sufficient to limit bacterial quantities in the intestine. Notably, both CM3 and acarbose-treated flies showed a reduced lifespan. However, this effect was absent in amylase inhibitor (AI) treated flies. Together, given α-glucosidase is a crucial requirement for disaccharide digestion, we suggest that inhibition of α-glucosidase by CM3 enhances disaccharide load in the distal gastrointestinal tract, thereby promoting intestinal bacteria overgrowth. However, it remains speculative if this here described former unknown function of CM3 might contribute to the development of gastrointestinal symptoms observed in NCWS patients which are very similar to symptoms of patients with small intestinal bacterial overgrowth.

**Graphical Abstract:**
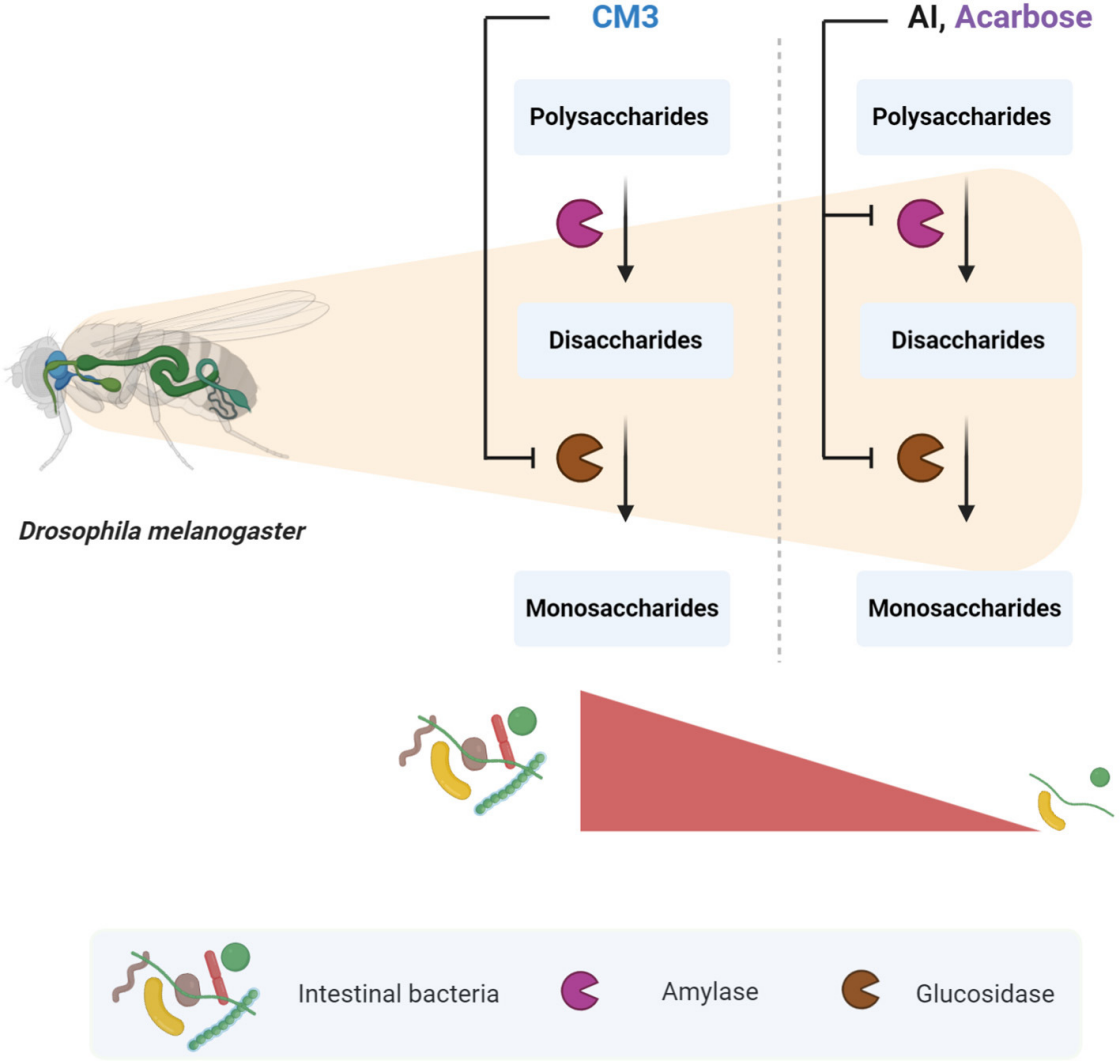
The effect of CM3 on carbohydrate digestive enzymes and intestinal bacteria in *Drosophila melanogaster*. Designed by Biorender.com.

## Introduction

*Triticum aestivum* (bread wheat) is one of the most widely consumed and cultivated wheat species due to its flexible growth requirements, health benefits and unique properties (1, 2). Multiple studies have correlated various disorders to the increased consumption of wheat (3–5). The most common of these conditions include wheat allergy (WA), celiac disease (CD), and non-celiac wheat sensitivity (NCWS) (6). These three disease entities are characterized by adverse reactions to wheat ingestion including gastrointestinal tract (GIT)-symptoms such as meteorism, abdominal pain and diarrhea. However, diagnostic markers like duodenal lesions, immunoglobulin E (IgE) or tissue transglutaminase antibodies are absent in NCWS patients (7).

Thus, the diagnosis of NCWS is based on exclusion criteria which hinders the exact determination of the global disease prevalence, that is suggested to be roughly between 0.6 and 6% according to self-reported data (8).

Gluten represents 70–75% of the total proteins of wheat grains (9) and has been suggested as a major contributor to NCWS pathogenesis alongside other wheat components such as fermentable, oligo, di, monosaccharides, and polyols (FODMAPs), amylase trypsin inhibitors (ATIs), and wheat-germ agglutinin (10).

ATIs are among a wide range of enzyme inhibitors that were evolved by plants to fight insect infestation (11). Nonetheless, they are capable of not only inhibiting insect digestive enzymes but also mammalian equivalents according to various studies (11, 12). It is estimated that their average consumption is between 0.5 and 1 g per day (6). Based on molecular weight, ATIs are categorized into three subclasses of proteins with a molecular mass of 12, 24, and 60 kDa. While the smallest group (12 kDa) contains monomeric proteins like 0.28, the 24 kDa group consists mainly of dimeric ATIs like 0.19 (13). The largest subgroup (60 kDa) consists mainly of the tetrameric CM proteins which are known for their solubility in chloroform and methanol (14). Non-covalently linked dimeric and tetrameric ATIs are stabilized by disulfide bonds which are important for their biological activity (14, 15). O'Connor and McGeeney were among the first to characterize wheat tetrameric inhibitors (16). They identified a protein with a molecular weight of 63 kDa which displayed strong inhibitory activity against human amylase (16). Further, it has been reported that proteins CM2, CM3, and CM16 are components of the tetrameric inhibitors CM proteins which constitute 50% of ATIs in *Triticum aestivum* (17, 18). Tundo et al. investigated the capacity of three ATI proteins, CM3, CM16, and 0.28 to trigger basophil degranulation against human sera of patients with WA (19). CM3 was the most potent allergen of all three inducing the highest release of β-hexosaminidase (19). Furthermore, allergenicity was decreased in transgenic lines obtained from the bread wheat cultivar Bobwhite silenced in three ATI genes CM3, CM16, and 0.28 (20). Kusaba-Nakayama et al. have demonstrated that certain wheat ATIs subunits specifically bind to IgE using sera from atopic dermatitis patients (4). Other studies suggested the involvement of TLR4 signaling pathway in ATI-induced intestinal inflammation (15, 21).

ATIs are reported to inhibit α-amylase (22). As such, they might interfere with digestion of carbohydrates which includes not only pancreatic enzymes like amylase but also glucosidases that are located at the brush border of the intestinal epithelium (23). Carbohydrate digestion is a complex process that involves various enzymes along the gut. Polysaccharide digestion is initiated by salivary α-amylase that hydrolyzes the α-(1→ 4)-D-glycosidic bonds, resulting in oligo or disaccharides (24). As food bolus passes through the duodenum, oligo-disaccharides are further broken down by pancreatic α-amylase (24). Terminal non-reducing α-1→ 4 linkage of oligosaccharides or disaccharides is hydrolyzed by α-glucosidases (23).

*Drosophila melanogaster* has gained increased attention as a valuable model in food and nutrition research. The *Drosophila* genome encompasses versatile digestive enzymes involved in the metabolism of carbohydrates, proteins and lipids (25). *Drosophila* and human intestines share remarkable resemblance in their digestive systems (26).

Thus, in order to better understand the pathophysiology of NCWS and the role of ATI, we aimed to recombinantly produce the most abundant ATI, CM3 (27) and study its function *in vitro* and in an *in vivo* model system utilizing *Drosophila melanogaster*.

## Materials and Methods

### Plasmid Construction

Recombinant CM3 protein was coded by the plasmid pcDNA3.1-CM3-His-C. The sequence of CM3 (AY436554 from *Triticum aestivum*), obtained from the website “National Center for Biotechnology Information” (www.ncbi.nlm.nih.gov), was *de novo* synthesized by Thermo Fisher Scientific (Waltham, MS, USA). To facilitate purification, the sequence for 6 histidine amino acids (His-Tag) and a C-Tag (EPEA) were added at the C-terminus. The sequence for CM3 followed by His- and C-Tag was cloned into pcDNA3.1 via *Kpn*I and *Bam*HI restriction sites, resulting in the plasmid pcDNA3.1-CM3-His-C.

### Expression and Purification of Recombinant CM3

LentiX 293T cells (Takara Bio Europe, Saint-Germain-en-Laye, France) were transiently transfected with the plasmid pcDNA3.1-CM3-His-C using calcium phosphate method. Cells with a density of 1-2 × 10^6^ were resuspended in DMEM high glucose (Thermo Fisher Scientific, Waltham, MS, USA), 10% (v/v) fetal bovine serum (Thermo Fisher Scientific, Waltham, MS, USA), and 1% (v/v) Penicillin-Streptavidin (Biowest, Nuaillé, France). They were seeded in 10 cm petri dishes and incubated at 37°C, 5% CO_2_ overnight. On the following day, cold transfection buffer was added dropwise to the dishes. To prepare the transfection solution for 20 dishes, 400 μg pcDNA3.1-CM3-His-C plasmid, 2.5 M calcium chloride and 100 mM chloroquine were mixed and filled up to 20 ml with H_2_O. This mixture was added slowly to 20 ml of 2x HBS (50 mM HEPES, 280 mM NaCl, 1.5 mM NaH_2_PO_4_, pH 7.05) with permanent injection of air via 5 ml pipette. After 8–12 h of incubation the media with transfection solution was discarded and fresh medium was added. Culture medium was fully exchanged every 1–2 days until cells were completely separated from the petri dish. Supernatant was mixed 1:10 with 10x His-buffer (500 mM disodium phosphate, 1.5 M sodium chloride, 100 mM imidazole, pH 8) and incubated at 4°C for at least 2 days, sterile-filtered and stored at 4°C until protein purification. Then, 1 L of supernatant was mixed with 2 ml Ni-NTA Matrix (Qiagen, Hilden, Germany) and kept overnight at 4°C. The mixture was subsequently added to a 10 ml polyprep column (Bio-Rad Laboratories, Munich, Germany). The matrix was washed two times with 10 ml cold His wash buffer, while the flow-through was collected as wash 1 and 2. To elute the protein CM3-His-C from the matrix, 3 ml of His-elution-buffer (50 mM disodium phosphate, 300 mM sodium chloride, 250 mM imidazole, pH 8) was added for 5 min and flow through was collected as elution 1. Subsequently, additional 2 ml elution buffer was added 1–4 times to get the elution fractions 2–5. To check remaining protein in the flow-through fractions and to confirm protein in elution fractions, a small aliquot was tested via SDS-PAGE and a western blot. If remaining protein could be detected in flow-through fractions the samples were pooled and purification procedure was repeated.

Elution fractions containing CM3, confirmed via western blot, were pooled and dialyzed against PBS. Pooled elution fractions were transferred to a dialysis membrane with 3.5 kDa molecular weight cut off (Carl Roth, Karlsruhe, Germany). The volume of PBS was about 100 times of the sample volume. Dialysis was performed three times for 6–16 h against PBS under slow stirring at 4°C. Subsequently, the protein concentration was increased by centrifugation for 10 min at 757 × *g* and 4°C using Vivaspin 5 kDa centrifugal concentrators (Sartorius, Göttingen, Germany).

### SDS-PAGE

Analysis of purified proteins was performed via sodium dodecyl sulfate-polyacrylamide gel electrophoresis (SDS-PAGE) using Bio-Rad (Munich, Germany) products. Protein samples were mixed with 4x SDS-buffer and heated for 5 min at 95°C to denature the proteins. An electrophoresis chamber was filled with electrophoresis buffer and a Criterion TGX polyacrylamide gel was installed. Samples were loaded and the gel was run for ~1 h at 240 V.

### Protein Staining With Colloidal Coomassie

Next, the gel was either be used for western blotting or protein staining using colloidal coomassie staining solution (10% (v/v) phosphoric acid, 0.8 M Ammonium sulfate, 20% (v/v) methanol, 1.5 mM Coomassie G-250). Proteins separated on a polyacrylamide gel were stained over night with colloidal coomassie staining solution. The following day, the gel was washed with H_2_O until the blue background was removed and bands got visible. Subsequently, the gel was documented by scanning or photographing.

### Western Blot

To detect specific proteins, they were separated on a polyacrylamide gel and blotted onto a PVDF-membrane (Bio-Rad Laboratories, Munich, Germany) with the Trans-Blot Turbo Transfer System (Bio-Rad Laboratories, Munich, Germany) according to the manufacturer's instructions. The membrane was incubated with blocking solution (3% (w/v) bovine serum albumin (BSA) in TBS for 1 h. Subsequently, the primary antibody anti-Penta-His (1:2,000) (Qiagen, Hilden, Germany) was added and incubated over night at 4°C under gentle shaking. On the next day, the membrane was washed three times with T-TBS for 10 min. The secondary antibody anti-mouse-IgG-HRP was added to the membrane and incubated for 1 h followed by triple washing with T-TBS for 10 min. Bands could be visualized by addition of a luminol containing substrate (Immobilon Western HRP Substrat, Merck, Darmstadt, Germany). Chemiluminescence was measured with ChemiDoc XRS+ system (Bio-Rad Laboratories, Munich, Germany).

### Automated Gel Electrophoresis

Experion automated gel electrophoresis was performed to measure the purity of CM3 according to the manufacturer's instructions (Bio-Rad Laboratories, Munich, Germany). Samples were analyzed under either reducing (by adding β-mercaptoethanol) or non-reducing conditions. The size of the proteins from the sample wells was calculated by using Pro260 ladder which is covering 10–260 kDa.

### Endotoxin Measurement

To detect contamination of CM3 protein with bacterial endotoxins, Pierce LAL Chromogenic Endotoxin Quantitation Kit (Thermo Fisher Scientific, Waltham, MS, USA) was used according to manufacturer's instructions. With this kit the amount of gram-negative bacterial endotoxins, which catalyze the activation of a proenzyme in the modified Limulus Amebocyte Lysate (LAL), can be determined. Activated proenzyme catalyzes the splitting of p-Nitroaniline (pNA) from the colorless substrate, which can be measured photometrically at 405–410 nm after stopping the reaction. The developed color intensity is proportional to the amount of endotoxin present in the sample and can be calculated in endotoxin units (EU) per ml using a standard curve. For the standard curve, E. coli endotoxin standards from 1 to 0.1 EU/ml were prepared. Three measurements of CM3 (70 μg/ml) and gliadin (320 μg/ml, Sigma-Aldrich, Steinheim, Germany) were performed and endotoxin levels were calculated.

### *In vitro* Trypsin Activity

The activity of bovine pancreatic trypsin (Sigma-Aldrich, Steinheim, Germany) was tested upon incubation with CM3. In short, 25 μl trypsin (85 U/ml) was mixed with 25 μl CM3 (1 μM), trypsin inhibitor from chicken egg white (ovoinhibitor, Sigma-Aldrich, Steinheim, Germany) or PBS in a 96- well plate and incubated for 30 min at 37°C under continuous shaking at 200 rpm. Ovoinhibitor was used as a positive control of trypsin inhibition. Benzoyl-L-arginine-p- nitroanilide (2.18 mg) (L-BAPA; Sigma-Aldrich, Steinheim, Germany) was dissolved in 100 μl dimethylsulfoxid (DMSO) and further diluted with 50 ml PBS resulting in the trypsin substrate solution (100 μM). One hundred microliter of trypsin substrate solution (100 μM) was added to the pretreated trypsin and incubated for 60 min at 37°C under continuous shaking. After stopping the reaction with 50 μl acetic acid (30%), OD was measured at the wavelength of 405 and 550 nm. Reference wavelength values were subtracted from values measured at 405 nm and average of the duplicate values was taken. For analysis, values of trypsin incubated with PBS was set as 100% activity.

### *In vitro* α-Amylase Activity

We investigated the effect of CM3 on amylase activity using α-amylase from human saliva (Sigma-Aldrich, Steinheim, Germany). At first, 10 μl of α-amylase (8 U/ml) were incubated for 15 min with 40 μl CM3 (2 μM), amylase inhibitor (AI) (2 μM) (A1520, Sigma-Aldrich, Steinheim, Germany), acarbose (1, 10, 100 μM) (Sigma-Aldrich, Steinheim, Germany) or 66.43 μg/ml BSA in PBS in a 96 well plate at 37°C under continuous shaking. Subsequently, 30 μl of starch (1.3 mg/ml) dissolved in water dH2O was added and the enzymes were given 15 min at 37°C to catalyze hydrolyzation of the glycosidic bonds. The reaction was stopped by the addition of 20 μl of 1 M HCl. One hundred microliter of lugol solution (diluted 1:40, Carl Roth, Germany) was added to detect remaining starch. The iodine from lugol solution forms an intermolecular charge-transfer complex with the starch resulting in a blue color. Absorbance was measured at 580 nm with Spectramax id3 microplate reader (Molecular Devices, CA, USA).

### *In vitro* Activity of α-Glucosidase

Effect of CM3 on α-glucosidase activity was measured by incubation of 10 μl of α-glucosidase from *Saccharomyces cerevisiae* (250 mU/ml) (Sigma-Aldrich, Steinheim, Germany) with 60 μl of CM3 (0.4 μM), AI (0.4 μM), acarbose (1.7 mM), or PBS for 30 min in a 96 well round bottom plate at 37°C while shaking at 200 rpm. After 15 min of incubation with 20 μl (2 mM) of the substrate 4-Nitrophenyl-α-D- glucopyranosid (Sigma-Aldrich, Steinheim, Germany) the reaction was stopped by addition of 50 μl 0,1 M Na_2_CO_3_. Hydrolyzation of the substrate by α-glucosidase results in a yellow color that can be detected by a photometer at 400 nm. Additionally, OD 600 nm was measured as reference and subtracted from the values taken at 400 nm. Average of the triplicates was taken, and activity of glucosidase was calculated assuming 100% activity with PBS.

### D*rosophila melanogaster in vivo* Model

#### *Drosophila melanogaster* Stocks and Diet

All reagents and materials were purchased from Genesee Scientific Corporation (San Diego, CA, USA) and Sigma-Aldrich (Steinheim, Germany) unless otherwise noted.

W^1118^ *Drosophila melanogaster* were maintained on 10% Caltech medium (CT) containing 5.5% dextrose, 3.0% sucrose, 6% corn meal, 2.5% inactive dry yeast, 1% agar, 0.3% nipagin, and 0.3% propionic acid in a climate chamber (HPP 750, Memmert, Schwabach, Germany) under the following standard conditions: a temperature of 25°C, relative humidity of 60% and 12-h day/night cycle. For all experiments, age-matched flies derived from synchronized eggs prepared according to the method of (28) were used. To investigate the effect of CM3 on fruit flies, standard medium (SM) consisting of 5% sucrose, 8.6% corn meal, 5% inactive dry yeast, 0.5% agar, 0.3% nipagin, and 0.3% propionic acid was supplemented with PBS, 2.6 μM recombinant CM3, 2.6 μM AI, or 2.6 μM acarbose. For sucrose-free experiments, sucrose-free SM was used.

#### Lifespan Experiment

Seventy-five male or female W^1118^ flies per treatment group were divided into three vials containing standard medium containing PBS, 2.6 μM CM3, 2.6 μM AI, or 2.6 μM acarbose. Flies were transferred to fresh medium every 2–3 days while dead flies were recorded. The experiment was repeated twice. In the second experiment, 100 flies per sex and treatment were used and separated into four vials.

#### Gustatory Assay

To exclude the potential effects of food intake, W^1118^ flies were fed SM supplemented with PBS, 2.6 μM CM3, 2.6 μM AI, or 2.6 μM acarbose for 5 days under standard conditions. Next, flies were transferred to SM stained with 0.2% w/v sulforhodamine B sodium salt (Sigma-Aldrich, Steinheim, Germany) under standard conditions for further 16 h. Following, 20 flies per treatment were homogenized in PBS plus 1% Triton X-100 with Ultra-Turrax T8 (IKA, Germany). Subsequently, 50 μl was transferred in duplicates to a 96-well plate and absorbance was measured at 550 nm with the Multiskan microplate reader (Thermo Fisher Scientific, Waltham, MS, USA).

#### Climbing Assay

The climbing ability of *Drosophila melanogaster* was tested as an indicator of overall fitness. After a 10-days treatment with PBS, 2.6 μM CM3, 2.6 μM AI, or 2.6 μM acarbose, 10 flies per sex and treatment were placed in an empty vial to record the flies' locomotor activity as reported earlier (29).

#### Triglyceride, Glucose, and Total Protein Analysis

After 10 days of treatment with PBS, CM3, AI, or acarbose, five W^1118^ flies per sex and treatment were homogenized in 250 μl PBS/Triton X-100 (1%, v/v). Lysates were centrifuged at 12,000 × *g* and 4°C for 5 min. Supernatants were stored at −80°C until further use. Triglyceride and glucose levels were measured by using commercially available kits (Fluitest TG and GLU; Analyticon Biotechnologies AG, Germany) according to manufacturer's instructions. Samples were diluted 1:2 and 1:5 with PBS/NaCl (0.9%, w/v) for triglyceride and glucose, respectively. Concentrations were calculated via the standard curve and normalized to median fly weights. Measurement of total protein was performed with Roti-Quant Universal (Carl Roth, Germany) according to manufacturer's instructions. The samples were diluted 1:30 with PBS. BSA dissolved in PBS was used as standard. Protein concentrations of the fly samples were calculated via the standard curve and normalized to fly weights.

#### Amylase Activity Assay

After 10 days of treatment, 13 W^1118^ flies from each treatment and sex groups were homogenized in 250 μl amylase assay buffer. Homogenates were centrifuged at 12,000 × *g* at 4°C for 10 min. The amylase activity assay (Sigma-Aldrich, USA) was performed according to the manufacturer's instructions. Values were normalized to the corresponding median total protein.

#### Isolation of Intestinal Bacteria From *Drosophila melanogaster*

Midguts of 5 flies per treatment group were dissected under the microscope in a small petri dish with 5% agarose or Sylgard 184 (Sigma-Aldrich, Steinheim, Germany) moisturized with PBS/DTT (0.016%, w/v) after 10 days treatment. Pooled guts were washed three times with PBS by flipping the reaction tube and spun down in a benchtop centrifuge. For hypotonic lysis of the intestinal cells, 500 μl of dH_2_O was added and guts were vortexed at 800 × *g* at RT for 30 min. Samples were centrifuged at 12,000 × *g* at 4°C for 3 min. The supernatant was discarded while bacteria and cell debris were dissolved in 100 μl PBS/glycerol (16%, v/v) and immediately frozen at −80°C.

#### Flow Cytometry

To analyze the bacterial load of the flies' intestinal tracts, the isolated bacteria were washed 2–3 times with ice-cold FACS-buffer (1x PBS containing 0.1% w/v /BSA and 0.1% w/v /NaN_3_) and centrifuged at 757 × *g* for 3 min at 4°C. Living bacteria were stained with 10 μM SytoGreen BC (Thermo Fisher Scientific, Waltham, MS, USA) on ice for 20 min. Bacteria were pelleted, supernatant was discarded, and the cell pellet was dissolved in 500 μl FACS-buffer. A total of 20,000 living bacteria were measured on the flow cytometry with Attune NxT (Thermo Fisher Scientific, Waltham, MS, USA).

### Statistical Analysis

Data are presented as mean ± SD unless otherwise indicated. Statistical analysis was performed with GraphPad Prism 6.0 (GraphPad Software, Inc., San Diego, CA). Significance was calculated with matched one-way ANOVA followed by Dunnett's test comparing sample with control. Differences between groups were considered to be significant at a *P*-value of ^*^ <0.05, ^**^ <0.01, ^***^ <0.001, or ^****^ <0.0001.

## Results

### Recombinant Expression and Purification of CM3

**Figure 1:**
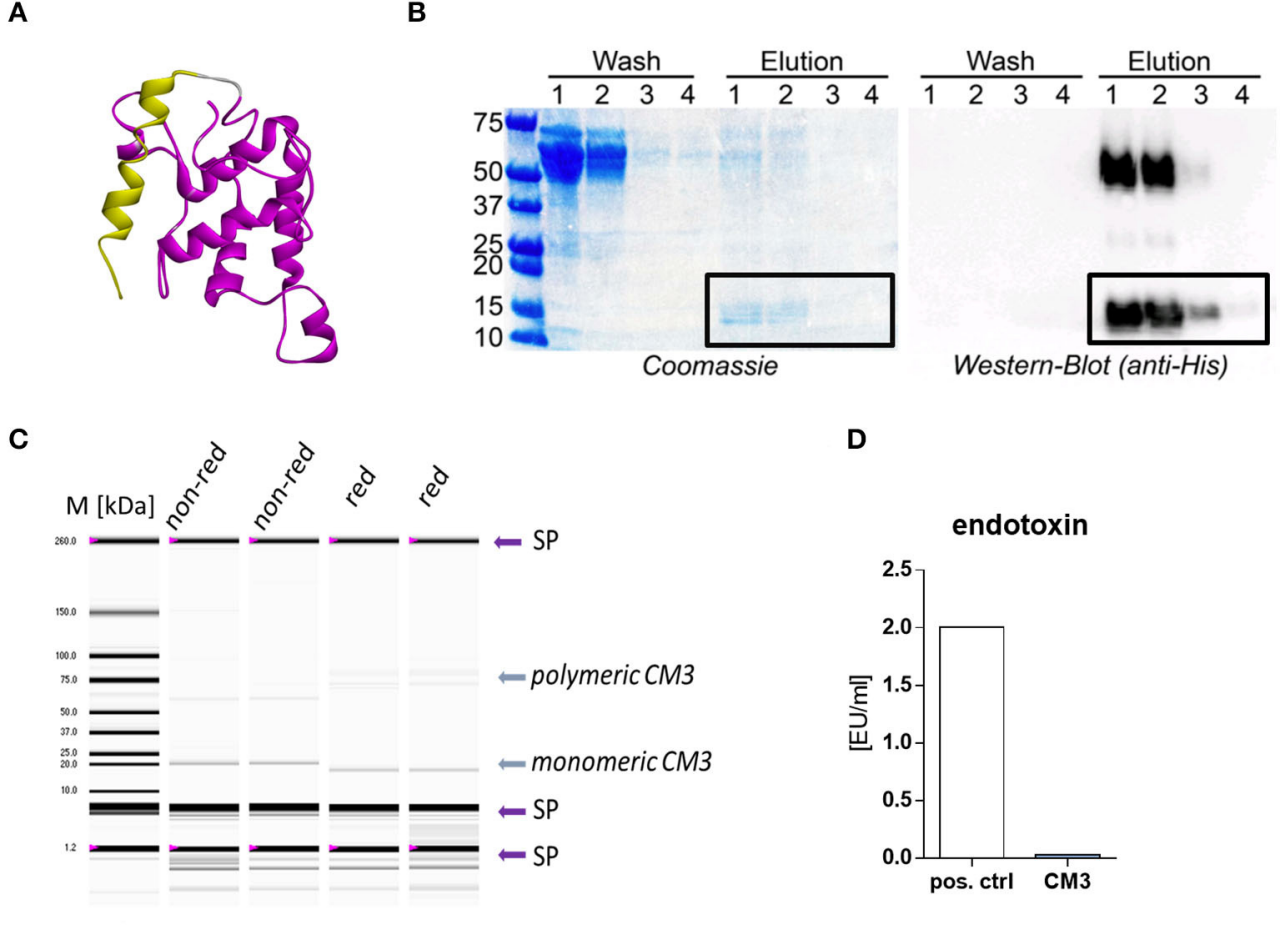
Recombinant expression and purification of CM3. **(A)** A structural model of the CM3 protein was modeled from the respective protein sequence using the PHYRE2 protein fold recognition server. CM3 protein domains were predicted using the InterPro server CM3 protein domains were predicted using the InterPro server (https://www.ebi.ac.uk/interpro/download/) and the SMART server (http://smart.embl-heidelberg.de). Yellow: amino acids (aa) 1–25 = signal peptide, Purple: aa 29–52 = PS00426, conserved site of cereal trypsin/alpha-amylase inhibitors family. **(B)** Coomassie stained SDS-gel (left) and western blot (right) of fractions collected during Ni-NTA affinity cleaning of CM3. Recombinant protein was detected with anti-His antibody. **(C)** Experion automated gel electrophoresis was performed to measure the purity of CM3 under non-reducing (n. red.) or reducing conditions (red.) in duplicates. Arrows with SP indicate technically necessary system peaks. Arrow with CM3 indicates CM3 protein at predicted size. **(D)** LAL assay was performed to measure endotoxin level. CM3 was tested directly after dialysis and gliadin was used as positive control (pos. ctrl). One endotoxin unit (EU) equals ~0.1–0.2 ng endotoxin.

We focused on chloroform-methanol soluble protein 3 (CM3) given that it is the most common ATI and is linked to several wheat related sensitivities (4, 15, 27). Homology model of CM3 protein shown in Figure 1A includes N-terminal signal peptide and the conserved site of cereal trypsin/alpha-amylase inhibitors family.

We generated lentiviral transfer plasmid pcDNA3.1-CM3-His-C that encodes the sequence of CM3 C-terminally tagged with a 6x-histidin tag and a C-tag. Subsequently, LentiX 293T cells were transiently transfected with the plasmid and supernatants were collected daily.

The Ni-NTA column purification was carried out to extract 6xHis-tagged CM3. Coomassie-stained SDS-PAGE analysis revealed the efficient removal of contaminating proteins after the first two washing steps (Figure 1B). The elution of 15 kDa target CM3 protein band was confirmed by western blot experiments utilizing an anti-His antibody. However, western blot showed evident 50 kDa band in the first two elution lanes (Figure 1B), pointing to polymeric CM3 proteins This band faded in the third elution and disappeared with the fourth elution. The total yield of CM3 ranged between 60 and 90 μg/ml from further dialysis of elution fractions containing CM3. Subsequently, an aliquot of purified CM3 was analyzed with a highly sensitive automated gel electrophoresis. The protein was tested in duplicates under reducing (red.) and non-reducing (n. red.) conditions (Figure 1C). As expected, a protein band at 20 kDa is visible in all lanes (Figure 1C).

Purified CM3 was free of endotoxins as determined by the limulus amebocyte lysate assay (LAL assay) with endotoxin levels <0.05 EU/ml in CM3 aliquots (Figure 1D).

### CM3 Protein Displays α-Glucosidase Inhibitory Activity

**Figure 2:**
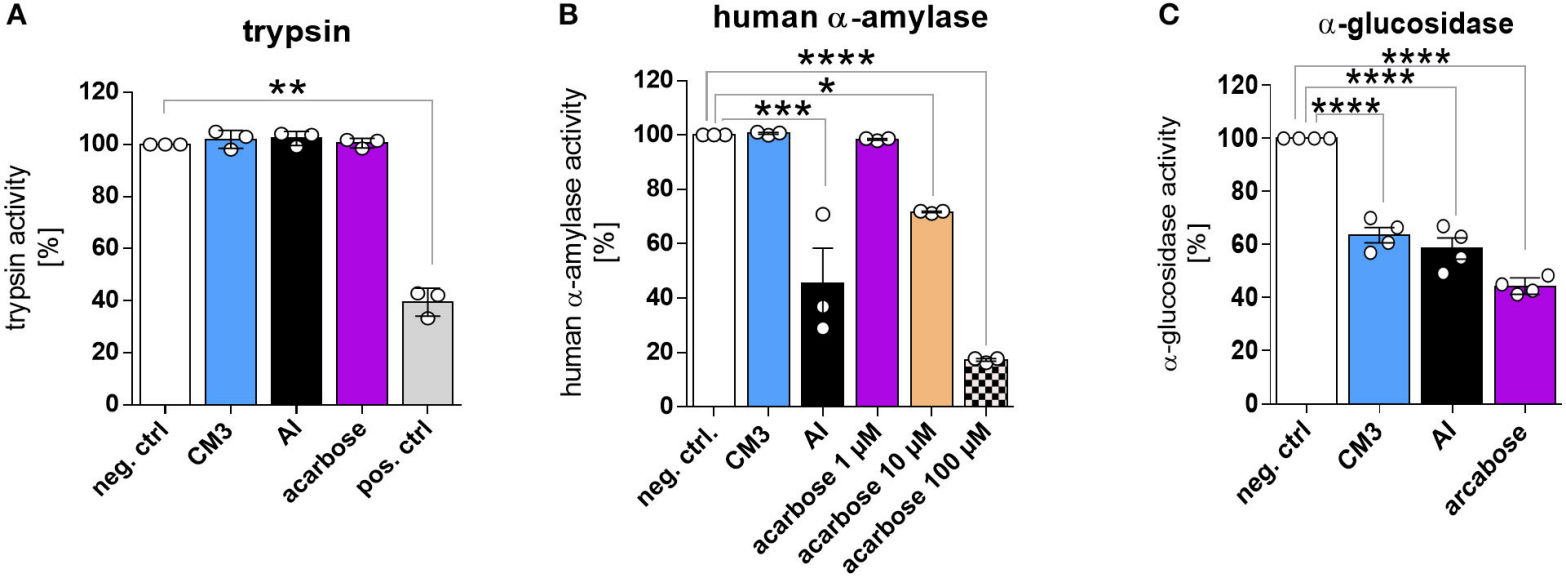
Effect of CM3 on digestive enzymes *in vitro*. **(A)** Effect of CM3 (2 μM), α-amylase inhibitor from *Triticum aestivum* (AI; 2 μM), or acarbose (2 μM) on trypsin activity was tested with bovine pancreatic trypsin. Positive control was trypsin inhibitor from chicken egg white (pos. ctrl) and PBS treated trypsin was used as a negative control (neg. ctrl). **(B)** Influence of CM3 (2 μM), AI (2 μM), or acarbose (1–100 μM) on amylase activity was measured utilizing salivary human α amylase. **(C)** Effect of CM3 (0.4 μM), AI (0.4 μM), or acarbose (500 μM) on α-glucosidase activity. Data are mean ± *SD*. All experiments were repeated at least three times. Significance was calculated with matched one-way ANOVA followed by Dunnett's comparing sample with control (*p ≤ 0.05, **p ≤ 0.01, ***p ≤ 0.001, ****p ≤ 0.0001).

ATIs can inhibit amylase and trypsin enzymes (13). Hence, we investigated the effect of CM3 on various digestive enzymes *in vitro*. CM3 did not exert any visible trypsin inhibitory potential as compared to ovoinhibitor, a known trypsin inhibitor (Figure 2A). Additionally, we could not observe any effect of CM3 on several concentrations of trypsin (data not shown). No effect was also detected upon incubation with either AI from *Triticum aestivum* or acarbose. We next examined the influence of CM3 on carbohydrate-hydrolyzing enzymes. CM3 failed to reduce human α-amylase activity (Figure 2B). On the other hand, both AI and acarbose showed human α-amylase inhibitory activity (Figure 2B). Higher concentrations of acarbose were required to exhibit strong human α-amylase inhibitory effect (Figure 2B). Additionally, a range between 0.015 and 1 μM of CM3 showed no effect on human α-amylase activity (data not shown). Furthermore, we examined the effect of CM3 on α-glucosidase. As acarbose inhibits α-glucosidase (23), it was included as a positive control for the assay. Compared to the PBS treated samples, CM3 decreased α-glucosidase activity by ~40% which was comparable to acarbose effect (Figure 2C). Surprisingly, AI showed comparable inhibitory effect as CM3 on α-glucosidase activity (Figure 2C).

### CM3-Treated Flies Showed Short Lifespan

**Figure 3:**
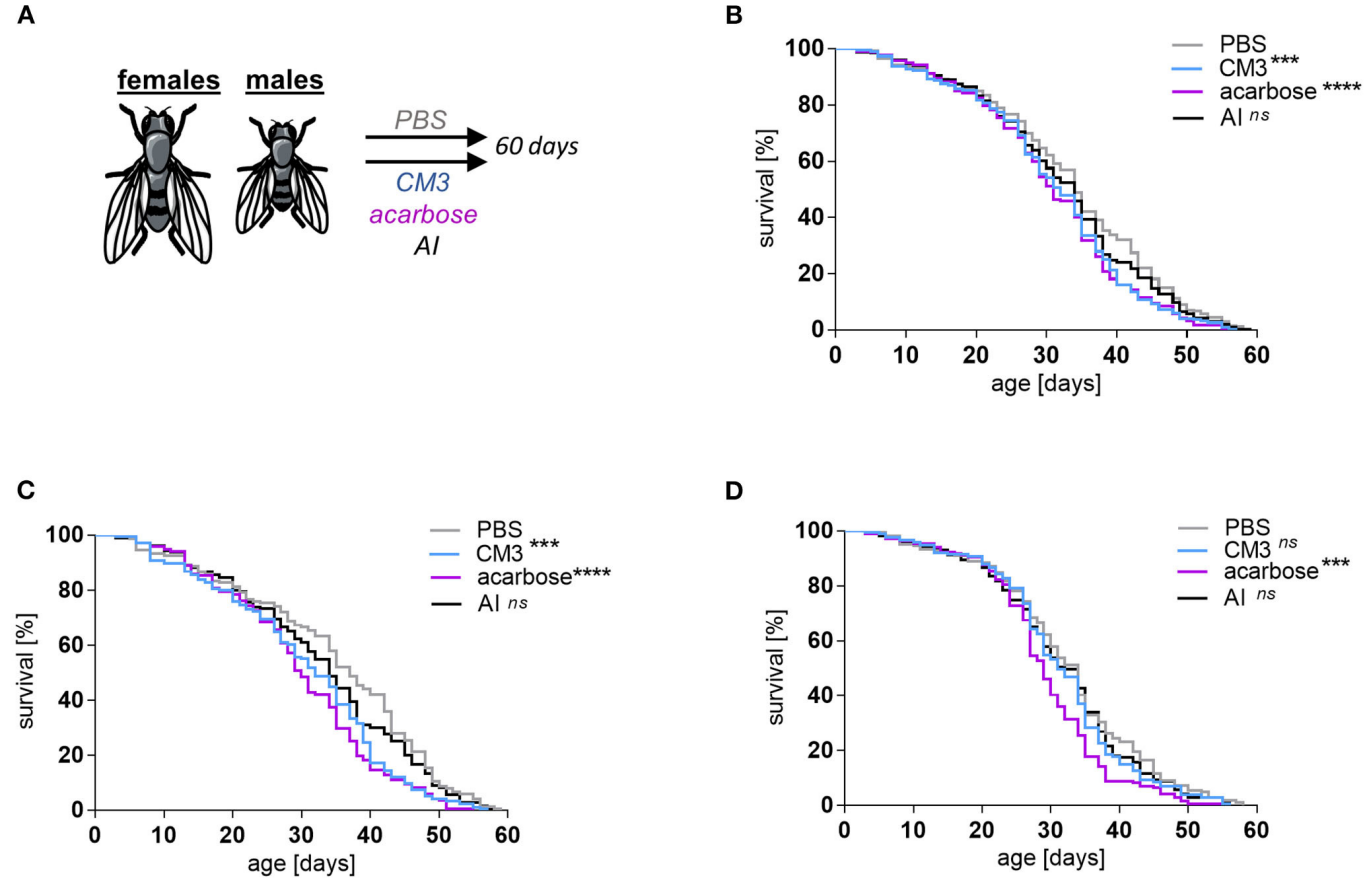
Effect of CM3 on the life expectancy of *Drosophila melanogaster*. (**A)** Schematic representation of longevity experiments in which female and male W^1118^ flies were treated with PBS, CM3, AI, or acarbose. Influence of CM3, acarbose or AI feeding on the survival rates of male and female flies combined **(B)**, female flies **(C)**, or male flies **(D)**. Data were recorded until the last fly died. Significant differences between treatments were calculated by applying the Log-rank (Mantel-Cox) test (ns: not significant, ***p ≤ 0.001, ****p ≤ 0.0001).

To investigate the α-glucosidase inhibitory capacity of CM3 in an *in vivo* model, we assessed CM3 effect on the lifespan of fruit flies. CM3, AI or acarbose were added to SM of W^1118^ at a concentration of 2.6 μM (Figure 3A). Supplementation with CM3 significantly shortened the lifespan of flies (Figure 3B). The difference between PBS and CM3 treated flies became evident from day 30 and reached its peak on day 40 (Table 1). This effect was stronger in female flies than their male counterparts (Figures 3C,D). However, AI did not significantly affect the lifespan of treated flies (Figures 3B–D). Notably, acarbose severely affected life expectancy in a greater extent than CM3 or AI (Figures 3B–D).

**Table 1:** Survival rates at indicated time points of fruit flies fed PBS, CM3, acarbose, or a α-amylase inhibitor (AI).

	**Day 10**	**Day 20**	**Day 30**	**Day 40**	**Day 50**	**Day 60**
PBS	94%	85%	62%	32%	7%	0%
CM3	93%	82%	54%	16%	4%	0%
Acarbose	95%	83%	51%	16%	3%	0%
AI	95%	83%	58%	24%	6%	0%

*The color value indicates major differences were detected on day 40*.

### High Amylase Activity Was Observed Upon CM3 Treatment

**Figure 4:**
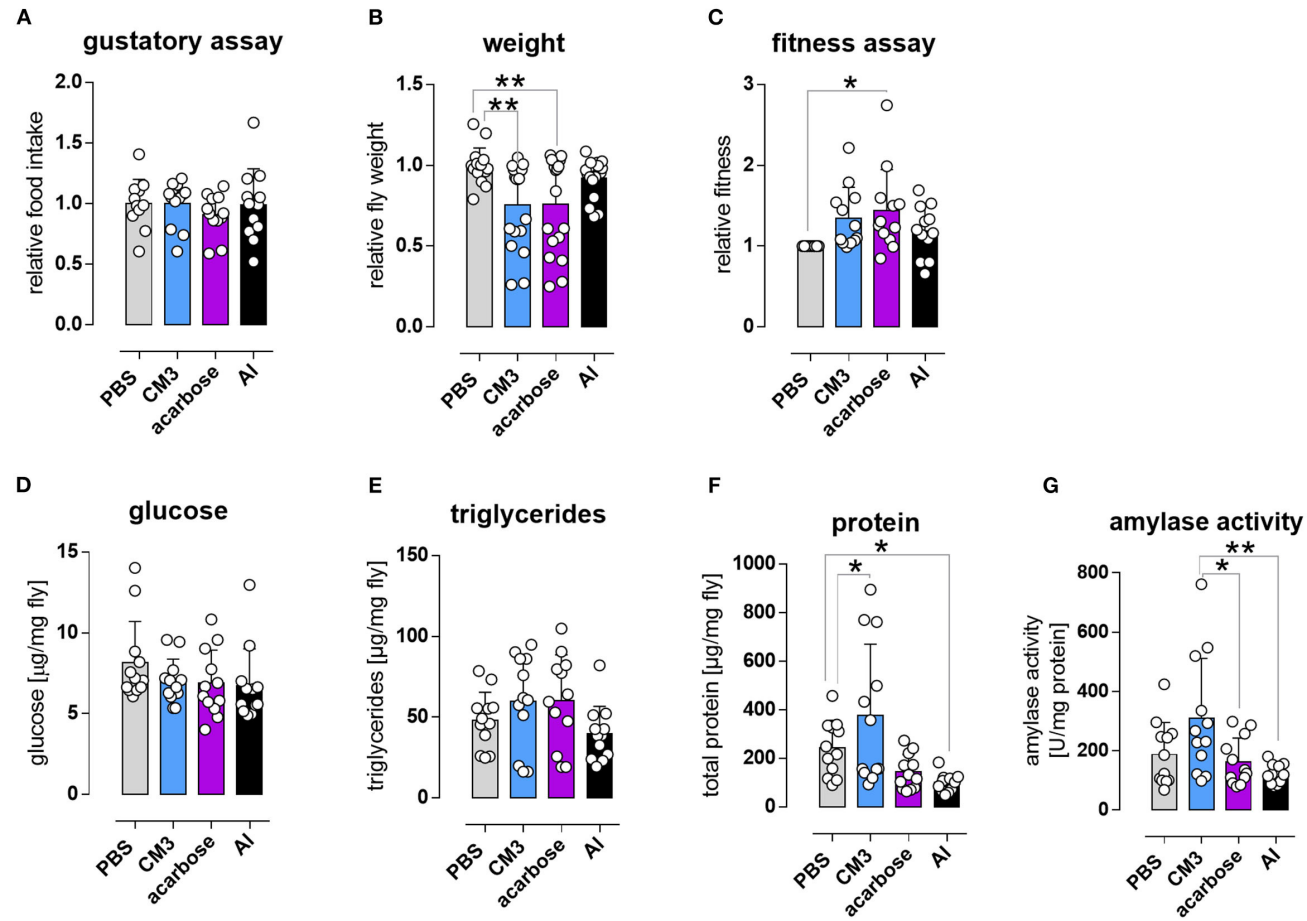
Short term effect of CM3 on *Drosophila melanogaster*. **(A)** Gustatory assay of W^1118^ flies treated with CM3, acarbose, AI, and PBS supplementation for 5 days. **(B)** Fruit flies were weighed after 10 days of CM3, acarbose, or AI treatment and compared to control (PBS) flies. **(C)** Relative fitness score of CM3, acarbose, or AI-treated flies was assessed by testing the climbing ability of these flies. Glucose **(D)**, triglycerides **(E)**, and protein **(F)** levels of treated flies were assessed using whole fly homogenates of five flies. **(G)** Relative amylase activity was measured in whole fly homogenates of 13 flies treated with PBS, CM3, acarbose, or AI. Data are mean ± *SD* (*p ≤ 0.05, **p ≤ 0.01).

To investigate whether the reduced lifespan in CM3, AI, or acarbose fed flies is a result of decreased food consumption, food intake and weight changes were monitored in W^1118^ flies treated with PBS, CM3, AI, or acarbose for 10 days. There was no difference of food intake between the groups (Figure 4A). Nevertheless, weight was significantly reduced in CM3 and acarbose fed flies (Figure 4B). Surprisingly, the climbing ability of acarbose fed flies was significantly enhanced in comparison with PBS fed flies, while no differences were observed in AI or CM3 fed flies (Figure 4C). Unexpectedly, glucose or triglyceride levels were not altered in flies under diets containing CM3, AI or acarbose (Figures 4D,E). Of note, total protein levels were significantly increased in CM3 fed flies compared to PBS fed flies, while AI fed flies displayed significantly decreased protein levels (Figure 4F). Lowest amylase activities were observed in flies on diets containing AI or acarbose, while highest amylase activity was detected under a CM3 containing diet (Figure 4G).

### CM3 Fed Flies Exhibited Increased Intestinal Bacterial Load

**Figure 5:**
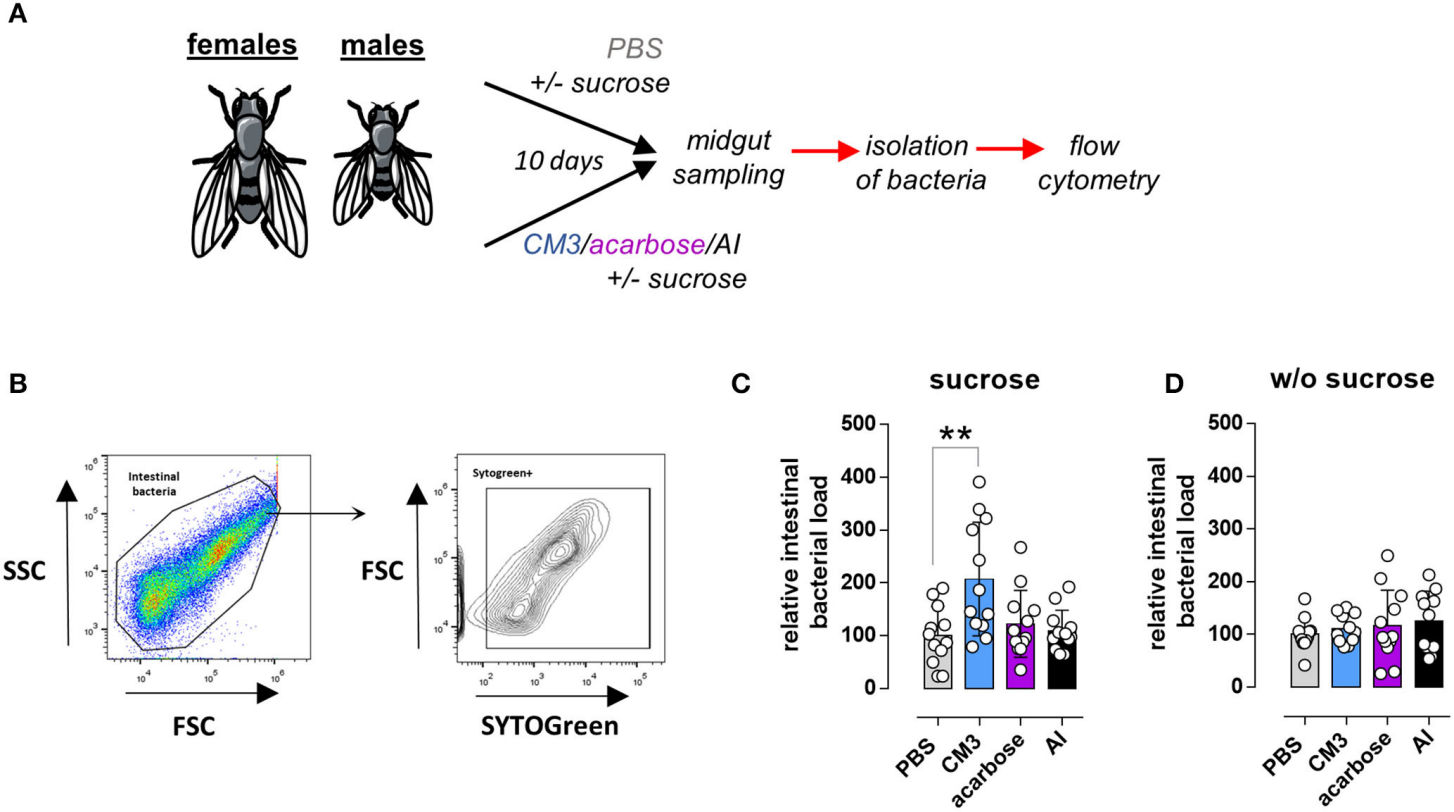
Effect of CM3 on intestinal bacteria isolated from *Drosophila melanogaster*. **(A)** Schematic representation of the experimental setup to study the effect of CM3, acarbose, or AI on intestinal bacteria. **(B)** Gating strategy to identify and quantify living intestinal bacterial. Bacterial density of flies treated for 10 days with either sucrose-rich **(C)** or sucrose-free **(D)** diet supplemented with CM3, acarbose, AI, or PBS. Data are presented as box graph. Lines within the boxes indicate median values; whiskers—min and max. Significance was calculated with matched One-way ANOVA followed by Fisher's LSD comparing samples with PBS control (**p ≤ 0.01).

To study the effect of CM3, AI, or acarbose consumption on intestinal microbiota count, isolated bacteria from the midguts of adult flies were stained with SytoGreen dye and quantified by flow cytometry (Figure 5A). The gating strategy and analysis were mainly based on the method described previously by our group (30). In short, the population of intestinal bacteria was identified based on the forward (FSC) and side scatter (SSC) (Figure 5B). SytoGreen positive events were defined as living bacteria. The measured events (bacteria) were then correlated to the corresponding median fly weight per each group. Total intestinal bacterial load from CM3 fed flies was significantly increased in comparison with control group (Figure 5C). Nonetheless, neither acarbose nor AI consumption showed any effect on the intestinal bacteria count in flies (Figure 5C). The observed CM3-mediated effect on the intestinal bacteria count was abolished when flies were fed a sucrose-free diet (Figure 5D). In these experiments, the overall bacterial load of CM3 fed flies was comparable to their control counterparts on a sucrose-free diet (Figure 5D).

## Discussion

CM3 is one of the most prevalent wheat ATI subunits (27). Accumulating evidence implicates CM3 in the pathogenesis of wheat-related hypersensitivity (4, 15, 19). However, little is known about the biological features of CM3 and its effects on carbohydrate hydrolyzing enzymes. In this study, we first produced CM3 protein based on its coding sequence from *Triticum aestivum* and examined its effect on lifespan, metabolism and intestinal bacteria in *Drosophila melanogaster*.

Here, we recombinantly generated and purified CM3 using column chromatography. Our results unexpectedly showed that, in contrast to the known amylase inhibitors such as AI from *Triticum aestivum* or acarbose, CM3 does not display human salivary α-amylase inhibitory activity. Terminal luminal digestion of disaccharides is undertaken by a wide range of small intestinal brush border enzymes including α-glucosidase (31). Notably, recombinant CM3 proved to inhibit α-glucosidase activity resembling the effect of AI or acarbose. The latter drug acarbose is a pseudo-tetrasaccharide and displays high binding affinity to α-glucosidases and lower binding affinity to α-amylase, thereby preventing binding and processing of natural ligands such as disaccharides. Hence, based on this function acarbose is frequently used in glycemic control of type 2 diabetes mellitus (32).

Surprisingly, in contrast to a previous report (33), CM3 did not exert inhibitory effect on bovine pancreatic trypsin. This might be attributable to the purity of extracted proteins from wheat used in the study from Mancinelli et al. that were comprised of CM2 and CM3 proteins. In other words, single CM3 is not capable of blocking trypsin activity but might be able to induce such effect in synergy with other ATI subunits.

In the present study, we utilized *Drosophila melanogaster* as a model organism to study the *in vivo* effects of CM3, given that it shares plenty of similarities with humans in terms of their digestive systems (34, 35). *Drosophila* amylases have been well-characterized at both genetic and molecular levels (36–38). Amylases are highly expressed and secreted into the midgut of the fly which corresponds to the mammalian small intestine (39). Our results show that amylase activity was increased in flies fed a sucrose and CM3 containing diet, while it was unaffected in flies that were fed a sucrose and AI or acarbose containing diet.

Based on these results, we hypothesized that the inability of host enzymes to digest disaccharides would result in an overgrowth of intestinal bacteria. Indeed, we detected an increase of the intestinal bacteria load in flies fed a sucrose and CM3 containing diet. Surprisingly, no alterations were observed in flies fed an AI or acarbose containing diet. These findings highlight the hypothesis that single α-glucosidase inhibition is more influential in contrast to the combined inhibition of α-amylase and α-glucosidase. This may be due to the generation of a higher disaccharide load under single α-glucosidase inhibitory conditions as α-amylase is still active after CM3 consumption. However, under conditions that block α-amylase and α-glucosidase such as consumption of acarbose or the here utilized AI from *Triticum aestivum*, starch is not digested to generate disaccharides anymore, leading to a lower disaccharide load in the gut. These findings support our previous *in vitro* experiments showing that CM3 acts mainly on glucosidases. Gastrointestinal side-effects have been reported with medications that competitively inhibit α-glucosidase (23). Symptoms that vary from flatulence to abdominal distention and diarrhea are caused by undigested and unabsorbed carbohydrate contents (40, 41). Patients with small intestinal bacterial overgrowth (SIBO) also demonstrate such symptoms (42, 43). SIBO is characterized by increased numbers of bacteria in the small intestine (44). These bacteria are mainly strict anaerobes that usually colonize the colon (43). Khazaei et al. showed how flexible gut microbiota could be in unfavorable growth conditions using two bacteria prominent in SIBO (45). The anaerobe *Bacteroides thetaiotaomicron* that possesses strong disaccharidase machinery thrived in the presence of oxygen and simple sugars consuming *Klebsiella pneumoniae* under normoxic condition (45). Although previous clinical trials reported a high occurrence of gastrointestinal side-effects such as flatulence, loose stools and/or abdominal discomfort after the consumption of acarbose due to high disaccharide load in the lower gastrointestinal tract (46), we did not find a bacterial overgrowth in acarbose fed fruit flies. Notably, gastrointestinal side-effects seem to mainly occur in the US population (47) but not or limited in the German (48, 49) or Asian (50, 51) populations, raising the hypothesis that differences in dietary behaviors (e.g., high vs. low disaccharide consumption) may critically influence the occurrence of these side-effects. Furthermore, gastrointestinal side-effects after acarbose consumption have been suggested to occur in a dose-dependent manner (52, 53).

Together, our novel findings demonstrate that CM3 possesses a unique functional α-glucosidase inhibitory property that distinguishes it from the other amylase inhibitors that display combined α-amylase and α-glucosidase inhibitory capacities. Based on the findings presented in this study, we speculate that patients under acarbose treatment should avoid the intake of CM3 rich wheat products. However, further dose-escalating studies are needed to elucidate the complex interplay between amylase inhibitors and CM3 in the context of carbohydrate digestion and potential harmful gastrointestinal side-effects.

## Data Availability Statement

The datasets presented in this study can be found in online repositories. The names of the repository/repositories and accession number(s) can be found in the article/supplementary material.

## Author Contributions

SDi, SDe, and CS: conceptualization. SDi, AEW, and SDe: methodology. A-LT, MR, AEW, and SDe: formal analysis. A-LT and MR: investigation and visualization. A-LT, MR, and SDe: writing/original draft preparation. AEW, SDi, SDe, and CS: writing—review and editing. SDe and CS: supervision and project administration. CS: funding acquisition. All authors have read and agreed to the published version of the manuscript.

## Conflict of Interest

The authors declare that the research was conducted in the absence of any commercial or financial relationships that could be construed as a potential conflict of interest.

## Abbreviations

AI: amylase inhibitor
ATIs: amylase trypsin inhibitors
BSA: bovine serum albumin
CD: celiac disease
CM3: chloroform/methanol soluble protein 3
FODMAPs: fermentable, oligo, di, monosaccharides and polyols
IgE: immunoglobulin E
LAL: limulus amebocyte lysate
NCWS: non-celiac wheat sensitivity
SDS-PAGE: sodium dodecyl sulfate-polyacrylamide gel electrophoresis
SIBO: small intestinal bacterial overgrowth
SM: standard medium
WA: wheat allergy.
